# Faceted-rough surface with disassembling of macrosteps in nucleation-limited crystal growth

**DOI:** 10.1038/s41598-021-83227-8

**Published:** 2021-02-12

**Authors:** Noriko Akutsu

**Affiliations:** Faculty of Engineering, Osaka Electro-Communication University, Hatsu-cho, Neyagawa, Osaka 572-8530 Japan

**Keywords:** Theory and computation, Scaling laws, Nonlinear phenomena, Phase transitions and critical phenomena

## Abstract

To clarify whether a surface can be rough with faceted macrosteps that maintain their shape on the surface, crystal surface roughness is studied by a Monte Carlo method for a nucleation-limited crystal-growth process. As a surface model, the restricted solid-on-solid (RSOS) model with point-contact-type step–step attraction (p-RSOS model) is adopted. At equilibrium and at sufficiently low temperatures, the vicinal surface of the p-RSOS model consists of faceted macrosteps with (111) side surfaces and smooth terraces with (001) surfaces (the step-faceting zone). We found that a surface with faceted macrosteps has an approximately self-affine-rough structure on a ‘faceted-rough surface’; the surface width is strongly divergent at the step-disassembling point, which is a characteristic driving force for crystal growth. A ‘faceted-rough surface’ is realized in the region between the step-disassembling point and a crossover point where the single nucleation growth changes to poly-nucleation growth.

## Introduction

Determining surface roughness is crucial for understanding many phenomena associated with crystal surfaces^1–13^. However, developing methods to measure surface roughness has proven to be not as straightforward as had been expected^14,15^. The roughness of a surface is defined by the variance of the surface height associated with the roughening transition on low Miller-index surfaces such as the (001) surface at equilibrium^14,15^. The roughening transition belongs to the Berezinskii–Kosterlitz–Thouless (BKT)^16–22^ universality class. Denoting the roughening transition temperature of the (001) surface as ${T_{\text{R}}}^{(001)}$, the (001) surface is rough for temperatures $T \ge {T_{\text{R}}}^{(001)}$, where the square of the surface width $W^2(L)$ is logarithmically divergent with respect to the linear system-size *L*. For $T<{T_{\text{R}}}^{(001)}$, the (001) surface is smooth and the surface width is constant and does not depend on the system size.

It has been believed that a faceted surface is necessarily smooth. The roughening transition is connected to the faceting transition at equilibrium^23–28^. For a small crystal droplet, the shape of the droplet with the least surface free energy is the equilibrium crystal shape (ECS), which is obtained by the Wulff construction^29,30^ or by the Andreev method^31–33^. On the ECS, the faceting transition occurs at the roughening transition temperature. The Gaussian curvature on the ECS is proportional to $W^2(L)/\ln L$ and the inverse of the determinant of the surface stiffness tensor^25^. Hence, for $T<{T_{\text{R}}}^{(001)}$, the Gaussian curvature of the (001) surface is zero, and the (001) surface then appears on the ECS as a facet. The ECS consists of facets of smooth surfaces and curved rough vicinal surfaces.

For “sharp” but rough surfaces at non-equilibrium, the self-affinity on a surface obtained by a symmetry principle argument can explain a wide range of surfaces or interface phenomena^34–39^. Here, a “sharp” surface means an atomically smooth surface locally. Hence, the surface height is well defined as *h*(*x*, *y*) at a site (*x*, *y*) on a 2D square lattice. Self-affinity is invariance under anisotropic scale transformations, in contrast to self-similarity, which is invariance under isotropic scale transformations. Using the surface height *h*(*x*, *y*), the surface width *W*(*L*, *t*) is defined by

1 $$\begin{aligned} W(L,t)=\sqrt{\langle [h(x,y)- \langle h(x,y) \rangle ]^2 \rangle }, \end{aligned}$$

where *t* is time and $\langle \cdot \rangle$ is an ensemble average. The surface width *W*(*L*, *t*) for a kinetically roughened surface is known to satisfy a Family–Vicsek scaling relation^35,36^:

2 $$\begin{aligned} W(L,t)\sim L^\alpha f(L^{-z}t), \ z=\alpha /\beta , \end{aligned}$$

where the $\alpha$, $\beta$, and *z* exponents are referred to as the roughness, growth, and dynamic exponents, respectively. In the non-equilibrium steady-state (in the limit $t \rightarrow \infty$), the surface width *W* becomes $W(L) \sim L^{\alpha }$. The theoretical values of $\alpha$ for a 2D surface in 3D are 0 and 0.386 for the BKT-rough and the Kardar–Parisi–Zhang (KPZ)-rough^40,41^ surfaces, respectively.

Although the Family–Vicsek scaling relation can explain many cases of algebraic divergence of surface or interface width, the KPZ exponent is rarely observed in crystal growth^36,41,42^. For example, a recent experiment on thin film growth of CdS^43^ showed a roughness exponent $\alpha$ of $0.78\pm 0.07$.

Faceted-like rocky and “rough” crystal shapes such as SiC^7^, Si^3–5^, or faceted-like dendritic shapes such as snowflakes^1^, are commonly observed crystal formations. The branching dendrites seen in snowflakes are caused by the Mullins–Sekerka (MS) instability^44^ in the thermodynamic scale. The tip velocity of a dendrite obeys a universal behaviour based on MS-instability^1,45^. Interestingly, the branching of the tip of a dendrite for a faceted surface also seems to obey a universal behaviour. However, the reason for the similarity between tip branching for a faceted shape and a rough surface has not been studied sufficiently.

To reproduce a faceted-like shape of a crystal for “diffuse” and rough surfaces, many phase-field models have been applied^46–49^. The most recent phase-field model and calculations on ice^49^ reproduced the 3D faceted shapes, including snowflakes. Here, a “diffuse” surface means an “atomically rough” surface, originating from a liquid–gas interface^21,50,51^. An example of a diffuse (atomically rough) but smooth surface is the (0001) surface of $^4$He crystals in superfluid helium^52^. The (110) surfaces of Ag$_2$Se and Ag$_2$S^53,54^ and the (100) and (111) surfaces of tetrabrommethane^55^ are considered to be examples of diffuse but globally smooth surfaces at lower temperatures than the roughening transition temperatures. Since the phase-field model applies for the mesoscopic scale, it reproduces the MS instability automatically under appropriate boundary conditions.

However, the problem with phase-field modelling is the connection between the phenomenological parameters in the basic equations of the phase-field model and the physical quantities based on the atomic surface structure^46–49^.

The aim of this article is to clarify whether a vicinal surface with faceted macrosteps can have a self-affine-rough structure in nucleation-limited crystal growth. We will show how sharp and faceted surfaces at equilibrium can roughen while keeping a faceted structure. The squared surface width is calculated using the Monte Carlo method on the vicinal surface tilted from the (001) surface to the (111) surface. To set up faceted macrosteps at equilibrium, the restricted solid-on-solid model with point-contact-type step–step attraction (p-RSOS model)^33,56–64^ is adopted (refer to the section “Methods”). The temperature is set in the step-faceting zone^59^, where the surface tension is discontinuous, and only the (001) surface and (111) surface are thermodynamically stable at equilibrium.

## Results

### Faceted-rough surface

**Figure 1:**
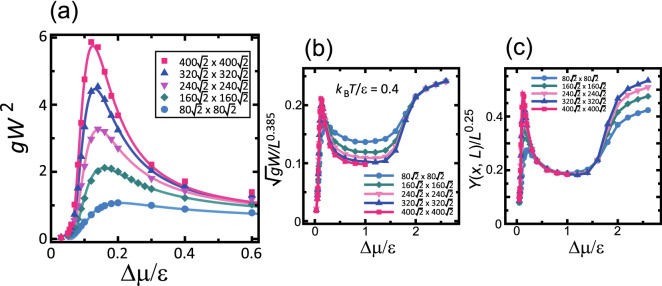
$\Delta \mu$ dependence of the square of the surface width $gW^2$, where $\Delta \mu$ is the driving force for crystal growth. *g* is $1+{\bar{p}}^2$, where ${\bar{p}}=3\sqrt{2}/8 \approx 0.530$ is the mean surface slope. $\epsilon _\text{int} / \epsilon = -0.9$. ${k_{\text{B}}T}/\epsilon =0.4$. $a=1$. (**a**) $gW^2$, with a maximum value at $\Delta \mu _R(L)$. The lines represent different sizes and are generated from Eqs. (6)–(12). (**b**) $\sqrt{gW^2}$ scaled by $L^{\alpha }$ with $\alpha =0.385$, which is the 2D KPZ roughness exponent in 3D. (**c**) *Y*(*x*, *L*) (Eq. (6)) scaled by $L^{\alpha '}$ with $\alpha ' =0.25$.

Figure 1a shows the $\Delta \mu$ dependence of $gW^2$ (Eq. (15)). $gW^2$ has a maximum at $\Delta \mu = \Delta \mu _R(L)$ (Table 1)^63^. The maximum value increases as the system size increases. $gW^2$ increases as *L* increases for $\Delta \mu _{co}^\text{(poly)}(L)< \Delta \mu < \Delta \mu _R(L)$, contrary to the expectation that $gW^2$ is independent of system size for $\Delta \mu < \Delta \mu _R(L)$. Here, $\Delta \mu _R(L)$ is the crossover point from a vicinal surface with (111) faceted macrosteps to a tilted surface with locally merged steps, and $\Delta \mu _{co}^\text{(poly)}(L)$ is the crossover point from single nucleation growth to poly-nucleation growth at the lower edge of a faceted macrostep^63^. In the region $\Delta \mu _{co}^\text{(poly)}(L)< \Delta \mu < \Delta \mu _R(L)$, the vicinal surface of the p-RSOS model grows in a step-detachment process in the manner of 2D poly-nucleation at the lower edge of a faceted macrostep^63,64^.

**Table 1:** Characteristic driving forces. $\epsilon _\text{int}/\epsilon =-0.9.$

	Value$/\epsilon$	Description
$\Delta \mu _y(L)$	0.016	Yielding point of the self-detachment of steps from a macrostep^63^
$\Delta \mu _{co}^\text{(poly)}(L)$	0.049	Crossover point from single 2D nucleation mode to 2D poly-nucleation mode^63^
$\Delta \mu _R(L)$	0.124	Transition point between the step-assembled phase and the step-disassembled phase^62,63^
$\Delta \mu _{co}^{(B-K)}$	0.3	Crossover point between BKT-rough surface and KPZ-rough surface ($\epsilon _\text{int}=0$, RSOS)^65^
$\Delta \mu _{kr}^{(001)}$	1.15	Kinetic roughening point for the (001) surface^65^

Surface slope ${\bar{p}}= 3\sqrt{2}/8 \approx 0.530$. $L=400\sqrt{2}a$ ($a=1$) for $\Delta \mu _y(L)$, $\Delta \mu _{co}^\text{(poly)}(L)$, and $\Delta \mu _R(L)$.

**Figure 2:**
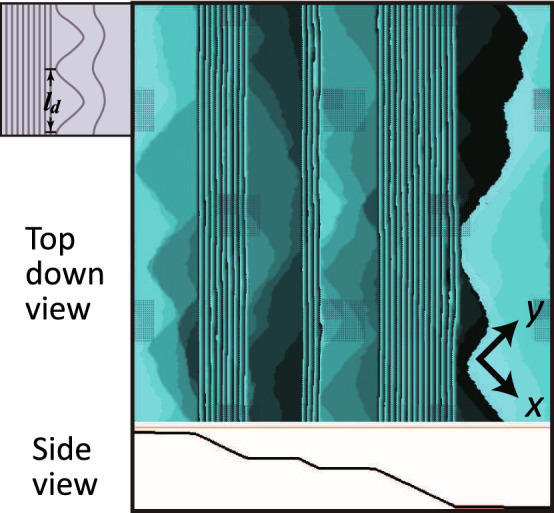
Typical morphology of a faceted-rough surface: snapshot generated by the Monte Carlo method at $4 \times 10^8$ MCS/site. ${k_{\text{B}}T}/\epsilon =0.4$. $\Delta \mu /\epsilon = 0.08$. $\epsilon _\text{int}/\epsilon =-0.9$. Size: $400 \sqrt{2} a \times 400 \sqrt{2}a$. $N_\text{step}= 300$. ${\bar{p}}=N_\text{step}a/L=3\sqrt{2}/8\approx 0.530$. $l_d$ (mean equal distance between 2D nuclei ^63^), $l_c$, and $t_d$ are 28*a*, 13*a*, and $1.44 \times 10^3$MCS/site (Eq. (4)). Inset: Illustration of a step-detachment mode in a poly-nucleation process at the edge of a faceted macrostep.

Figure 2 shows a typical morphology of a vicinal surface in the region $\Delta \mu _{co}^\text{(poly)}(L)< \Delta \mu < \Delta \mu _R(L)$ (see also Figs. S1b, and S2a,b). From the side view of the surface, we can see that the surface is covered with (111) side-surfaces and (001) terrace-surfaces. From the top view of the surface, islands with rounded triangle shapes are seen at the lower edge of the faceted macrosteps. Though the vicinal surface seems to be covered with smooth (001) and (111) surfaces, we can confirm that the surface grows continuously under the non-equilibrium steady state^63,64^. Hence, we call this surface structure for $\Delta \mu _{co}^\text{(poly)}(L)< \Delta \mu < \Delta \mu _R(L)$ the *faceted-rough* surface.

### Nucleation-limited continuous growth

**Figure 3:**
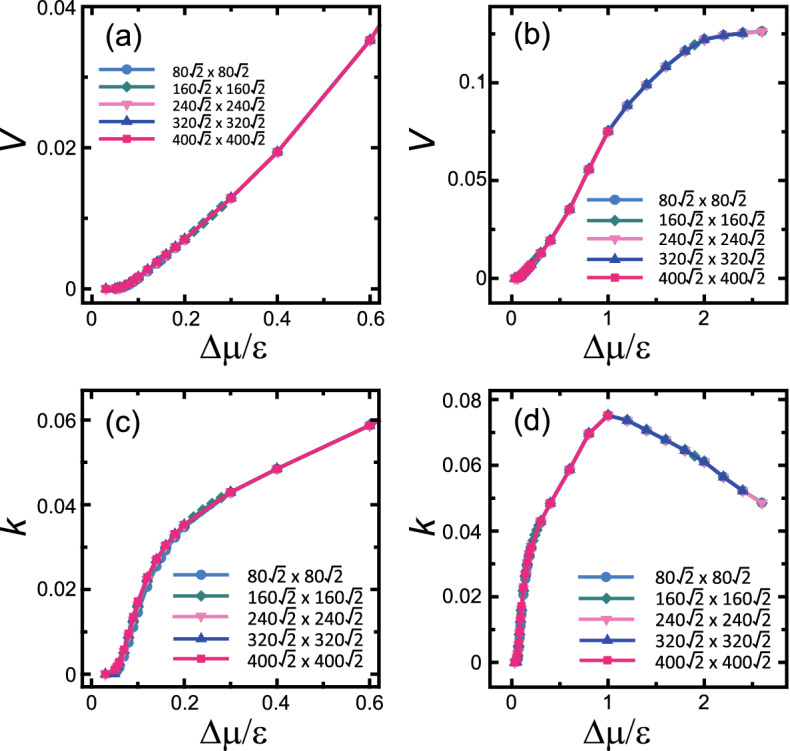
(**a**,**b**) Driving force dependence of the surface growth velocity. (**c**,**d**) Driving force dependence of the kinetic coefficient. ${k_{\text{B}}T}/\epsilon =0.4$. $\epsilon _\text{int}/\epsilon =-0.9$. $p=N_\text{step}a/L=3\sqrt{2}/8\approx 0.530$. $\theta =27.9$ °.

Figure 3 shows the driving force dependences of the surface velocity *V* and kinetic coefficient *k*, where $k = V \tau \epsilon / (\Delta \mu a$), $\tau =1$ is the time for an MCS/site, and $a=1$. In contrast to the surface width, *V* and *k* do not depend on the system size except at the region near equilibrium. *V* and *k* are similar to the original RSOS model for $\Delta \mu /\epsilon >1$ in the previous work^65^. This means that for $\Delta \mu /\epsilon >1$, steps are well separated in most cases (Figs. S1 and S2e,f). It should be noted that the critical nucleus sizes with a square shape are 2 and 1 for $\Delta \mu /\epsilon$ of 1 and 2, respectively. For $0.3< \Delta \mu /\epsilon <1.0$, *k* increases approximately linearly with respect to $\Delta \mu$, which gives $V \propto \Delta \mu ^2$. For $\Delta \mu _R(L)< \Delta \mu /\epsilon <0.3$ where the surface is rough with locally faceted macrosteps (Figs. S1 and S2c,d), *k* decreases rapidly as $\Delta \mu$ decreases.

In the region with a faceted-rough surface and for a rough surface with locally faceted macrosteps ($\Delta \mu /\epsilon < 0.3$), the vicinal surface grows in the 2D nucleation process at the lower edge of a faceted macrostep^63,64^. The mean height of a faceted macrostep $\langle n \rangle$ obeys the equation

3 $$\begin{aligned} \partial \langle n \rangle /\partial t = n_+ - n_-, \end{aligned}$$

where $n_+ = v_n p/a$ is the attachment rate of elementary steps to the faceted macrostep, $v_n$ is the growth velocity of an elementary step, *p* is the surface slope on a “terrace”, *a* is the lattice constant, and $n_-$ is the detachment rate of an elementary step from the faceted macrostep. $n_-$ is determined by the 2D poly-nucleation rate at the lower edge of the faceted macrostep^63,64^. At steady state, $\partial \langle n \rangle /\partial t=0$, and $n_+ = n_-$. The surface slope on a “terrace” is determined by $n_-$, so that the step-attachment rate balances the step-detachment rate.

To understand the poly nucleation rate near equilibrium, a characteristic length $$l_d$$ is introduced, representing the mean equal distance at which critical nuclei arise at the edge of a macrostep (inset in Fig. 2). The nuclei grow to merge with neighbouring nuclei after a time $$t_d$$, at which point a step detaches from the macrostep. The mean step-detachment time $$t_d$$, which is a characteristic time, is then expressed as^63,66^ $$ t_d=l_d/(2 v_t)=1/(I_n l_d), $$ where $$v_t \propto \Delta \mu $$ is the step zipping velocity and $$I_n$$ is the 2D nucleation rate at the step edge. Then, we obtain for $$0.05< \Delta \mu /\epsilon <0.15$$^63^

4 $$\begin{aligned} t_d= & {} \sqrt{\frac{C}{\sqrt{2} v_t Z}} \exp \{(g^*/2)/[\Delta \mu - \Delta \mu _y(L)]\}, \quad \frac{\sqrt{2}Z}{C} = c_k^2 k_\text{step}(\Delta \mu ), \quad c_k =0.604, \nonumber \\&k_\text{step}(\Delta \mu )=v_t \epsilon /\Delta \mu = 0.094+\exp [-5.8+ 0.18\epsilon /\Delta \mu ], \nonumber \\ V= & {} a/t_d, \quad l_d = 2v_t t_d, \end{aligned}$$

where $g^*/\Delta \mu = G(l_c)/{k_{\text{B}}T}$, $G(l_c)$ is the total step free energy of a critical nucleus at the macrostep-edge with critical size $l_c$, *Z* is the Zeldovich factor, *C* is a coefficient, $k_\text{step}(\Delta \mu )$ is the kinetic coefficient for an elementary step, *V* is the surface growth velocity, and *a* (=1) is the height of an elementary step. Here, $\Delta \mu _y(L)$ is a correction term introduced in our previous work^63^ to ensure that the surface velocity agrees with that obtained by the classical 2D nucleation theory. The Monte Carlo results are well reproduced by Eq. (4) for $0.05< \Delta \mu /\epsilon <0.15$^63^. When $\Delta \mu$ decreases and approaches $\Delta \mu _y(L)$, the surface growth velocity *V* decreases, whereas $t_d$ and $l_d$ increase, based on Eq. (4).

For $L>l_d$ and $t>t_d$, the vicinal surface grows continuously. While for $L<l_d$, the surface grows intermittently in the manner of a 2D single nucleation process at the macrostep edges due to the finite size effect (Fig. S1a). $\Delta \mu _{co}^\text{(poly)}$ is approximately estimated by

5 $$\begin{aligned} l_d(\Delta \mu _{co}^\text{(poly)}) \approx L. \end{aligned}$$

### Roughness exponents

Figure 1b shows the ratio of $\sqrt{g}W$ to $L^{0.385}$. The obtained results do not depend on the initial configuration. This is in contrast to the mean-height of a faceted macrostep $\langle n \rangle$, which is known to be sensitive to the history of the surface configuration^63,64^. As seen from Fig. 1b, the lines for $\Delta \mu /\epsilon > 1.8$ for different system sizes coincide. The power 0.385 is a universal value for the roughness exponent $\alpha$ for 2D KPZ-rough surfaces in 3D. Therefore, a vicinal surface for $\Delta \mu /\epsilon > 1.8$ is KPZ-rough.

In our previous work on the original RSOS model^65^, where $\epsilon _\text{int}=0$, the vicinal surface for $\Delta \mu /\epsilon > 1.8$ is shown to be KPZ-rough. Around $\Delta \mu /\epsilon \sim 1$, a broad peak is observed corresponding to the kinetic roughening point $\Delta \mu _{kr}^{(001)}/\epsilon$ for the (001) surface. However, such a peak is not observed for p-RSOS. Instead, there is a sharp peak at $\Delta \mu _R(L)$, which is less than $(1/5)\Delta \mu _{kr}^{(001)}$ of the RSOS model.

**Figure 4:**
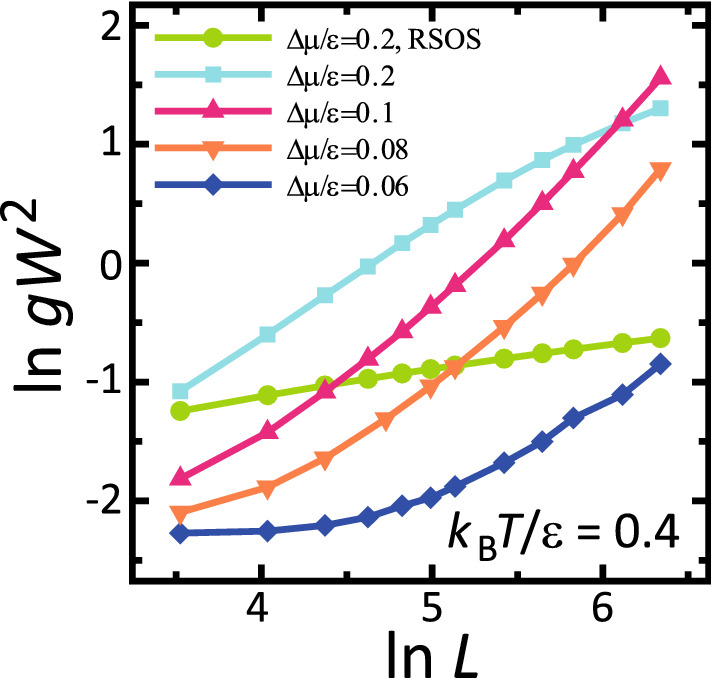
Size dependence of $gW^2$. ${k_{\text{B}}T}/\epsilon =0.4$. $\epsilon _\text{int}/\epsilon =-0.9$. ${\bar{p}} = 3\sqrt{2}/8 \approx 0.530.$ $a=1$.

For $\Delta \mu _R(L)< |\Delta \mu | < 0.4\epsilon$, $gW^2$ shows algebraic growth for lengths less than 200*a*. A typical case is shown in Fig. 4 for $\Delta \mu /\epsilon = 0.2$. At a small scale, $gW^2$ increases as $L^{0.95}$. The value of the exponent agrees with that for a zigzag structure of a 1D single step on a surface. Since relatively large locally merged steps remain, the value of the exponent indicates that the merged steps shift location in a “wiggly” manner, as if they were single steps.

At a scale larger than 200*a*, the slope changes. We will return to this point in the following subsection.

For the faceted-rough region $\Delta \mu _{co}^\text{(poly)}< \Delta \mu < \Delta \mu _R(L)$, $gW^2$ shows an approximate algebraic increase as the system size *L* increases for large *L* (Fig. 4). For large lengths $5.5<\ln L <7$, the Monte Carlo results were fitted to $\ln gW^2=2 \ln w_0 + 2\alpha _p \ln L$ by the least square method. The obtained values are listed in Table 2. Irrespective of temperature, $\alpha _p$ has values between 0.58 and 0.79. These values are about twice the KPZ-universal value of 0.386. Hence, the roughness of a faceted-rough surface is larger than the roughness of the original RSOS model. For large *L*, the vicinal surface is approximately self-affine. It is interesting that many observed values on the vicinal surface at nanometer scale are similar to those of the roughness exponent^36,41^. In a real system, a length $400\sqrt{2}a$ with $a \sim 4 \AA$ corresponds to about 230 nm.

**Table 2:** Roughness exponent $\alpha$ and provisional roughness exponent $\alpha _p$.

${k_{\text{B}}T}/\epsilon =0.4$			${k_{\text{B}}T}/\epsilon =0.2$		
$$\Delta \mu /\epsilon $$	$$\alpha _p$$	$$w_0/a $$	$$\Delta \mu /\epsilon $$	$$\alpha _p$$	$$w_0/a$$
0.06	0.58	0.016	0.2	0.71	0.022
0.08	0.78	0.010	0.25	0.72	0.041
0.1	0.77	0.017	0.275	0.68	0.059
0.12	0.67	0.036	0.3	0.59	0.11
$\Delta \mu _R^*/\epsilon$	$\alpha$	−	$\Delta \mu _R^*/\epsilon$	$\alpha$	−
0.090	0.60	−	0.27	0.59	−

$$240\sqrt{2}a \le L \le 400\sqrt{2}a$$. $$\epsilon _\text{int}/\epsilon =-0.9.$$ $${\bar{p}} = 3\sqrt{2}/8 \approx 0.530$$.

As shown in Fig.  4, the roughness depends on the length scale (refer to the following subsection). For relatively small $\Delta \mu$, this change of roughness can be seen clearly. For $\Delta \mu /\epsilon = 0.06$, where $l_d=125a$ and $t_d=6.7 \times 10^3$ [MCS/site], the vicinal surface is smooth for lengths less than $l_d/2$ (Fig. S1a). While *L* increases to over $l_d$, $gW^2$ increases algebraically. The slope of the lines becomes slightly steeper as *L* increases. Therefore, we conclude that faceted-rough surfaces consist of faceted structures at lengths less than $l_d/2$, whereas faceted-rough surfaces have approximately self-affine structures larger than $l_d$.

### Scaling function for step-disassembly

To explain why the provisional roughness exponent $\alpha _p$ gradually changes, we further analyse the driving force dependence of the surface width.

We assume that $gW^2$ is expressed by the following equation:

6 $$\begin{aligned} gW^2= gW^2(p_1)+Y^2(x,L), \end{aligned}$$

where $gW^2(p_1)$ represents the contribution from the “terrace” between the faceted macrosteps with slope $p_1$, $Y^2(x,L)$ represents the contribution from step-disassembling/assembling of the macrosteps, and *x* is an inverse-driving-force distance derived from the maximum value of $gW^2$ as $x=\epsilon /\Delta \mu - \epsilon /\Delta \mu _R(L)$.

At equilibrium, $p_1=0$; whereas $p_1$ increases as $\Delta \mu$ increases when $\Delta \mu$ exceeds a characteristic value $\Delta \mu _y(L)$. This is because an elementary step detaches from the lower edge of a faceted macrostep periodically on average (Eq. (4)). The slope dependence of $gW^2(p_1)$ at ${k_{\text{B}}T}/\epsilon =0.4$ is expressed by^65^:

7 $$\begin{aligned} gW^2(p_1) = [\ln (L/a)](A + B \ln p_1)^2, \quad A=0.319, \ B= 0.0650 . \end{aligned}$$

It should be noted that *A* and *B* for ${k_{\text{B}}T}/\epsilon =0.2$, 0.4, and 1.7 at equilibrium are the same within errors, as stated in Ref.^65^.

The $\Delta \mu$ dependence of the surface slope $p_1$ is obtained by the following equation^63^:

8 $$\begin{aligned} p_1 = \frac{c_p}{\sqrt{|\Delta \mu /\epsilon |}} \exp \left[ \frac{-g^*_p/2}{|\Delta \mu /\epsilon |-\Delta \mu _{yp}(L)/\epsilon } \right] , \end{aligned}$$

where $c_p$, $g^*_p$, and $\Delta \mu _{yp} (L)$ are fitting parameters. It should be noted that the set of values $\{g^*, \Delta \mu _{y}(L) \}$ in Eq. (4) and Table 1 were obtained by fitting Monte Carlo data to Eq. (8) in the fitting region $0.05< \Delta \mu /\epsilon < \Delta \mu _R(L)/\epsilon \approx 0.14$^63^. To describe the $\Delta \mu$ dependence of $p_1$ in the range $0.055 \le \Delta \mu /\epsilon \le 0.4$, the Monte Carlo data is re-fitted to Eq. (8), giving $c_p=0.357$, $g^*_p = 0.294$, and $\Delta \mu _{yp} (400 \sqrt{2})=0.026 \epsilon$.

**Figure 5:**
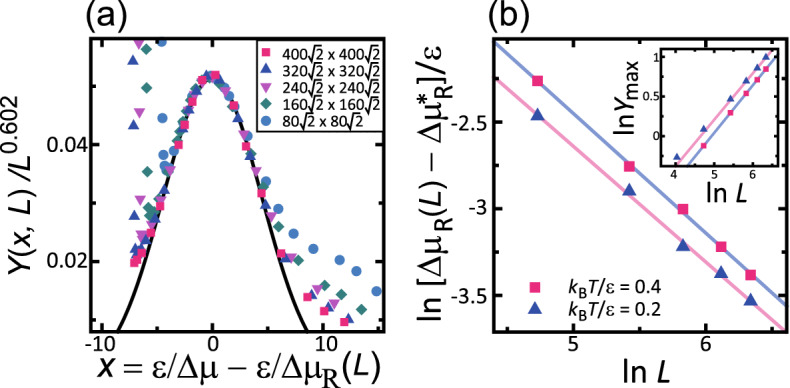
${k_{\text{B}}T}/\epsilon =0.4$. $\epsilon _\text{int}/\epsilon =-0.9$. ${\bar{p}} = 3\sqrt{2}/8 \approx 0.530.$ $a=1$. (**a**) Scaling function for step-disassembly. *Y*(*x*, *L*) follows Eq. (6) with Eqs. (7) and (8). The line follows Eq. (10). (**b**) Log–log plot of the linear system size *L* and the difference between $\Delta \mu _R$ and $\Delta \mu _R^*$, where $\Delta \mu _R^*$ is $\Delta \mu _R$ in the limit $L \rightarrow \infty$. Inset: log–log plot of $Y_{\text{max}}$ and *L*, where $Y_{\text{max}}$ is the value *Y*(0, *L*) (Eqs. (11) and (12)). Blue triangles and pink line: ${k_{\text{B}}T}/\epsilon =0.2$. Red squares and light blue line: ${k_{\text{B}}T}/\epsilon =0.4$.

We found that $Y^2(x,L)$ has a maximum at $\Delta \mu _R(L)$, and *Y*(*x*, *L*) is approximated by a Gaussian function for $\Delta \mu _{co}^{(poly)}< \Delta \mu /\epsilon < 0.4$ (Fig. 5), as follows:

9 $$\begin{aligned} Y(x,L) = Y_{{\text{max}}}(L) \exp [-B'x^2]. \end{aligned}$$

We introduce a scaling function ${\mathscr {Y}}(x)$ such that:

10 $$\begin{aligned} {\mathscr {Y}}(x)= & {} A'' \exp (-B'x^2) , \end{aligned}$$

11 $$\begin{aligned} Y_{{\text{max}}} (L)= & {} A''(L/a)^\zeta , \end{aligned}$$

12 $$\begin{aligned} \Delta \mu _R(L)/\epsilon= & {} (\Delta \mu _R^* + A'(L/a)^{- \chi })/\epsilon . \end{aligned}$$

The values of $A'$, $A''$, $B'$, $\Delta \mu _R^*$, $\zeta$, and $\chi$ are listed in Table 3.

**Table 3:** Scaling parameters.

	${k_{\text{B}}T}/\epsilon =0.4$	${k_{\text{B}}T}/\epsilon =0.2$
$\zeta$ ($= \alpha$)	0.60	0.59
$\chi$	0.69	0.67
$A'$	2.7	2.0
$A''$	0.051	0.065
$B'$	0.025	1.26

$$\epsilon _\text{int}/\epsilon =-0.9$$. $${\bar{p}} = 3\sqrt{2}/8 \approx 0.530.$$ Eqs. (9)–(12).

The shape of ${\mathscr {Y}}(x)$ is shown in Fig. 5a by a black line. The Monte Carlo data and ${\mathscr {Y}}(x)$ agree well around $|x|= \epsilon |1/\Delta \mu - 1/\Delta \mu _R(L)|<5$. For $x>5$, the surface grows in the single nucleation mode. Hence, the data strays from the line of ${\mathscr {Y}}(x)$. Since the change to the single nucleation mode is a finite size effect, the data for the smaller size begins to deviate for smaller *x* from the scaling function. For $x<-5$, faceted macrosteps disassemble to become locally merged steps.

The power law behaviours of $\Delta \mu _R(L)$ (Eq. (12)) and $Y_{\text{max}}$ (Eq. (11)) are shown in Fig. 5b. The data agree well with the lines. From the slope of the lines, we obtained $\zeta$ and $\chi$. The powers $\zeta$ and $\chi$ at different temperatures agree well.

The lines calculated using Eq. (6) for $\Delta \mu$ and *L* with Eqs. (9)–(12) are shown in Fig. 1. The lines reproduce the Monte Carlo results well for $\Delta \mu _{co}^{(poly)}< \Delta \mu /\epsilon <0.4$. For ${k_{\text{B}}T}/\epsilon =0.2$, $gW^2$ is similar to the case for ${k_{\text{B}}T}/\epsilon =0.4$. It is interesting that the values of $\chi$ and $\zeta$ are similar for the cases of ${k_{\text{B}}T}/\epsilon =0.4$ and 0.2. This suggests that the step-disassembling phenomenon around $\Delta \mu _R$ is a universal phenomenon. In addition, the point $\Delta \mu _R^*$ is a candidate for the non-equilibrium phase transition point.

Unexpectedly, as seen from Fig. 1c, *Y*(*x*, *L*) calculated by Eq. (6) from the Monte Carlo data shows algebraic divergence with respect to *L* with a roughness exponent $\alpha ' = 0.25$. The $Y(x,L)/(L/a)^{0.25}$ line for each size agrees well with the others for $0.4{\mathop {\sim }\limits ^{<}}\Delta \mu /\epsilon {\mathop {\sim }\limits ^{<}}1.6$. If $\sqrt{g}W$ is simply divided by $(L/a)^{0.25}$, the $gW^2$–$\Delta \mu$ lines do not coincide with each other. The value $\alpha '=0.25$ is the same as that for the tracer diffusion on a 1D lattice^67^. However, the physical connection between the tracer diffusion and the present case is unclear. This is left as a problem for a future study.

$\zeta$ agrees with the roughness exponent $\alpha$ in the limit $L \rightarrow \infty$. The maximum of $Y^2(x,L)$ diverges with exponent $L^{2\zeta }$ in the limit $L \rightarrow \infty$ (Eq. (11), Table 2). For $L \rightarrow \infty$ around $\Delta \mu _R(L)$, $gW^2$ increases asymptotically as $gW^2 \sim L^{2\zeta }$. This also means that $\alpha _p \rightarrow \zeta$ for $L \rightarrow \infty$. Therefore, $\zeta$ is the roughness exponent $\alpha$ in the limit $L \rightarrow \infty$.

For the *L* dependence of $\alpha _p$ around $\Delta \mu _R(L)$, since $\text{d}Y(x,L)/Y(x,L) \approx [\zeta +2A'B'x \chi (L/a)^{- \chi }\ (\Delta \mu _R(L)/\epsilon )^{-2} ]\text{d}L/L$ from Eqs. (9)–(12), we obtain

13 $$\begin{aligned} \alpha _p \approx \alpha + x[ 2A'B' \chi (L/a)^{- \chi }] (\Delta \mu _R^*/\epsilon )^{-2} + {\mathscr {O}}((L/a)^{- 2\chi }) . \end{aligned}$$

The second term in the right-hand side of Eq. (13) indicates the contribution from the shift of $\Delta \mu _R(L)$. For $\Delta \mu < \Delta \mu _R(L)$ with $x>0$, $\alpha _p$ becomes larger than $\alpha$. However, for $\Delta \mu _R(L) < \Delta \mu$ with $x<0$, $\alpha _p$ becomes smaller than $\alpha$.

## Discussion

The results for faceted-rough surfaces can explain why the giant Naica gypsum (CaSO$_4 \cdot$ 2H$_2$0)^9–11^ has a euhedral shape with a large size and high transparency. From laser confocal differential interference contrast microscopy (LCM-DIM) and atomic force microscopy (AFM) observations^10^, the gypsum surface was found to grow by a 2D nucleation process at the microscopic scale. The giant planar {010} surfaces of the crystal faces were found to consist of a hillock structure at the mesoscopic scale. The side surface of a hillock is a (100) surface, which grows very slowly. Such a slow growth is realised close to equilibrium. The surface is near equilibrium and the size of the critical nucleus is large, and therefore the time between formation of individual nuclei is long. Hence, inhomogeneity of inclusions should occur, which would cloud the crystal.

The continuous growth for the faceted-rough region in this work is possible for $\Delta \mu ^{(poly)}(L) <\Delta \mu$, where $\Delta \mu ^{(poly)}(L)$ is given by Eq. (5). Physically, $\Delta \mu ^{(poly)}(L)$ can be interpreted as a smaller limit of the driving force for poly-nucleation at the lower edge of a faceted macrostep. When the size is larger, $\Delta \mu ^{(poly)}(L)$ decreases. As pointed out by Alexander et al.^10^, 2D nucleation at the “valley” of the hillock, which is a concave line on the surface and corresponds to the lower edge of a faceted macrostep, has a lower activation energy than 2D nucleation on the {010} terrace surface. A similar 2D nucleation from the lower edge of a macrostep is observed experimentally for diamond^68^. These observed 2D nucleations at the lower edge of a macrostep are consistent with the present Monte Carlo results for a faceted-rough surface. Since the activation energy for 2D nucleation is significantly smaller than that on a terrace, continuous growth is possible. This contributes to keeping the inclusions homogeneous.

In addition, since the roughness exponent is $\alpha =0.6 <1$, *W*/*L* converges to zero in the limit $L \rightarrow \infty$. This means that the hillocks have an approximate self-affine structure, while the hillocks’ area converges to zero in a large length limit. Therefore, the giant gypsum has a euhedral shape and high transparency.

The faceted-rough surface in the present study may provide a connection between the strong anisotropic parameters in the phase-field modellings and the parameters for the atomic scale. The p-RSOS model has sharp surfaces and the height *h*(*x*, *y*) is well defined. However, when a surface is rough, the location of the surface becomes ambiguous. Hence, we consider the mean height of the surface and the variance of the height of the surface, which is divergent with respect to the system size *L*.

At equilibrium, $W^2/\ln L$ asymptotically equals the inverse of the determinant of the surface stiffness tensor^25^ of the BKT rough-surface in the limit $L \rightarrow \infty$. For a smooth surface, since $W^2/\ln L$ converges to zero in the large *L* limit, the determinant of the stiffness tensor is divergent, and the Gaussian curvature on the ECS should be zero.

At non-equilibrium in the faceted-rough region, if we regard the amplitude $w_0 =(W /L^{\alpha _p})$ as a local degree of roughness, we can explain the anisotropy of the local roughness of the faceted-rough surface. In the faceted-rough region, $w_0$ is 0.01–0.03, as seen from Table 2, whereas $w_0$ for a non-faceted vicinal surface is 0.08^65,69^. This anisotropy in $w_0$ is consistent with the anisotropies which were phenomenologically assumed^47–49^.

The results in the present study are consistent with the phenomena observed for Si melt–solid interfaces. In the case of Si melt growth, faceted dendrites^5^ similar to a rough surface or a faceted saw-like shape larger than 10 μm in length are known to appear on a vicinal interface during fast crystal growth, whereas the vicinal interface is planar for slow crystal growth^3^. Studies on Si melt growth have shown that the faceted plane is close to the (111) surface. Hence, the faceted surface was considered to be smooth and the surface grows in the 2D nucleation process. Nevertheless, the faceted saw-like shape was shown to be formed by MS instability^44,45^ by the observation of a negative temperature gradient before the solid–liquid interface^4^. The MS instability should be applicable to a rough surface^45^. This observed conflict may be solved by the present study. In the present work, we showed that the interface can be rough while maintaining a self-affine faceted macrostep structure near equilibrium with a 2D poly-nucleation process at the macrostep edges. Further experimental studies are expected.

## Conclusions

- In the faceted-rough region, a surface is smooth and faceted on the small scale, whereas at large scales, surfaces are rough and statistically self-affine with a roughness exponent of $\alpha =0.60$.
- The surface width *W* has a maximum at $\Delta \mu _R(L)$, where $\Delta \mu _R(L)$ is the step disassembling point and *L* is the linear size of the system. The maximum value of *W* diverges as $L^{\zeta }$ with $\zeta =0.60 \pm 0.02$. $\zeta$ agrees with $\alpha$ in the limit $L \rightarrow \infty$. $\Delta \mu _R(L)$ is much closer to the equilibrium than the kinetic roughening point of the terraces.
- $\Delta \mu _R(L)$ converges to $\Delta \mu _R^*$ in the limit $L \rightarrow \infty$. $\Delta \mu _R^*$ is a candidate for the non-equilibrium phase transition point.
- A faceted-rough vicinal surface is realized for $\Delta \mu _{co}^\text{(poly)}(L)< \Delta \mu < \Delta \mu _R(L)$, where $\Delta \mu _{co}^\text{(poly)}(L)$ is the crossover point between the 2D single nucleation mode and the successive poly-nucleation mode at the lower edge of a faceted macrostep. In this region, the provisional roughness exponent is $0.58 {\mathop {\sim }\limits ^{<}}\alpha _p {\mathop {\sim }\limits ^{<}}0.79$.

## Methods

###  p-RSOS model

The surface energy of a vicinal surface around the (001) surface is expressed by the following discrete Hamiltonian:

14 $$\begin{aligned} \mathcal{H}_\text{p-RSOS}&= \mathcal{N}\epsilon _\text{surf}+ \sum _{n,m} \epsilon [ |h(n+1,m)-h(n,m)| +|h(n,m+1)-h(n,m)|] \nonumber \\ & \quad +\sum _{n,m} \epsilon _\text{int}[ \delta (|h(n+1,m+1)-h(n,m)|,2) +\delta (|h(n+1,m-1)-h(n,m)|,2)] - \sum _{n,m} \Delta \mu \ h(n,m), \end{aligned}$$

where *h*(*n*, *m*) is the surface height at site (*n*, *m*), ${\mathscr {N}}$ is the total number of lattice points, $\epsilon _\text{surf}$ is the surface energy per unit cell on the planar (001) surface, and $\epsilon$ is the microscopic ledge energy. The summation with respect to (*n*, *m*) is taken over all sites on the square lattice. The RSOS condition, in which the height difference between the nearest neighbouring sites is restricted to $\{ 0, \pm 1\}$, is required implicitly.

The third and fourth terms in the right-hand side of Eq. (14) represent the point-contact-type step–step attraction. Here, $\delta (a,b)$ is the Kronecker delta and $\epsilon _\text{int}$ is the microscopic point-contact-type step–step interaction energy. $\epsilon _\text{int}$ contributes to the surface energy only at the collision point of neighbouring steps where the height difference can be $\pm 2$. When $\epsilon _\text{int}$ is negative, the step–step interaction becomes attractive (sticky steps). Quantum mechanically, $\epsilon _\text{int}$ is regarded as the energy gain by forming a bonding state between the dangling bonds at step edges at the collision point of neighbouring steps.

The fifth term in the right-hand side of Eq. (14) represents the driving force for crystal growth. Here, $\Delta \mu$ is $\mu _\text{ambient}- \mu _{crystal}$, where $\mu _\text{ambient}$ and $\mu _{crystal}$ are the bulk chemical potentials in the ambient phase and the crystal, respectively. At equilibrium, $\Delta \mu =0$; for $\Delta \mu >0$, the crystal grows, while for $\Delta \mu <0$, the crystal shrinks. Explicitly, $\Delta \mu$ is expressed by ${k_{\text{B}}T}\ln P/P_{eq}$ for an ideal gas and by ${k_{\text{B}}T}\ln C/C_{eq}$ for an ideal solution, where ${k_{\text{B}}}$ is the Boltzmann constant, *T* is temperature, *P* is vapour pressure, $P_{eq}$ is the vapour pressure at equilibrium, *C* is the solute-concentration, and $C_{eq}$ is the solute-concentration at equilibrium. If $P/P_{eq}$ or $C/C_{eq}$ is expressed by $1+ \sigma _\text{sat}$, where $\sigma _\text{sat}$ is the super saturation, $\Delta \mu \approx {k_{\text{B}}T}\sigma _\text{sat}$ for $\sigma _\text{sat}<<1$.

The p-RSOS model is a coarse-grained model relative to the model for first-principle quantum mechanical calculations, but it is a microscopic model relative to the phase-field model. In the p-RSOS model (Eq. (14)), the step–step attraction $\epsilon _\text{int}$ is the origin of the discontinuous surface tension. The surface energy $E_{\text{surf}}$ corresponds to the surface *free*-energy, which includes entropy originating from lattice vibrations and distortions^70^. $\epsilon$ and $\epsilon _\text{int}$ may soften due to lattice vibrations as the temperature increases. Hence, $E_{\text{surf}}$, $\epsilon$, or $\epsilon _\text{int}$ may slightly decrease as the temperature increases. However, $E_{\text{surf}}$, $\epsilon$, and $\epsilon _\text{int}$ are assumed to be constant throughout the work because we concentrate on studying the size and driving force dependence of the surface roughness.

### Mean surface slope and discontinuous surface tension

The mean surface slope tilted towards the $\langle 111 \rangle$ direction ${\bar{p}}$ is determined to be ${\bar{p}}=N_\text{step}a/L$, where $N_\text{step}$ is the number of elementary steps and *a* is the lattice constant. The partition function of the vicinal surface with slope ${\bar{p}}$ is obtained from ${\mathscr {Z}}= \sum _{h(\varvec{{x}},t)} \exp [ - \mathcal{H}/{k_{\text{B}}T}]$ with fixed $N_\text{step}$. The surface free energy $f({\bar{p}})$ is obtained from $f({\bar{p}}) = -{k_{\text{B}}T}\ln {\mathscr {Z}}$, and the surface tension $\gamma ({\bar{p}})$ is obtained from $\gamma ({\bar{p}})= f({\bar{p}})/\sqrt{1+{\bar{p}}^2}$.

Our previous studies at equilibrium showed that the p-RSOS model has a discontinuous surface tension^33,56–59,61^ at low temperatures with respect to the surface slope. The faceting diagram corresponding to the connectivity of the surface tension is obtained by calculating the partition function using the density-matrix renormalization group (DMRG) method. For $T<T_{f,1}$, the surface tension near the (111) surface becomes discontinuous. For $T<T_{f,2}$, which we refer to as the step-faceting zone^59^, only the surfaces with (001) and (111) are thermodynamically stable at equilibrium^57^. Hence, the vicinal surface with mean slope ${\bar{p}}$ becomes covered in hillocks with (001) terrace-surfaces and (111) side surfaces.

###  Monte Carlo method

The vicinal surface between the (001) surface and the (111) surface is considered using the Monte Carlo method with the Metropolis algorithm. The external parameters are temperature *T*, $\Delta \mu$, number of steps $N_\text{step}$, and the linear size of the system *L*. Atoms are captured from the ambient phase to the crystal surface, and escape from the crystal surface to the ambient phase. The number of atoms in a crystal is not conserved. Details of the Monte Carlo calculations are given in Ref.^69^. Figure 2 shows a snapshot of the vicinal surface.

### Mean surface height and surface width

The square of the surface width *W* for a tilted surface in the non-equilibrium steady state is defined by^69^

15 $$\begin{aligned} gW^2= & {} (a/L)\sum _{\tilde{x}}\langle [h(\tilde{x}, \tilde{y}, t) - (a/L)\sum _{\tilde{y}} h(\tilde{x}, \tilde{y},t) ]^2 \rangle , \nonumber \\ g= & {} (1 + p_x^2 + p_y^2) = 1/\cos ^2 \theta , \quad p_x = p_y= N_\text{step}a\sqrt{2}/L, \end{aligned}$$

where $\tilde{x}$ and $\tilde{y}$ represent a site on the surface along the $\langle 110 \rangle$ and $\langle \bar{1}10 \rangle$ directions, respectively, $\langle \cdot \rangle$ is the time average, *g* is the determinant of the first fundamental quantity of a curved surface^25^, and $\theta$ is the tilt angle inclined towards the $\langle 111 \rangle$ direction from the $\langle 001 \rangle$ direction. The time average is taken over $2 \times 10^8$ Monte Carlo steps per site (MCS/site), discarding the first $2 \times 10^8$ MCS/site^69^.

In our previous study on the surface width for the original RSOS model^65^, we found that the kinetic roughening point of the (001) surface $\Delta \mu _{kr}^{(001)}$ is different from the crossover point between the BKT-rough surface and the KPZ-rough surface $\Delta \mu _{co}^{B-K}$ (Table 1) with respect to the driving force for crystal growth $\Delta \mu$. For high $\Delta \mu$, the vicinal surface of the RSOS model near the (111) surface is KPZ-rough; whereas the vicinal surface of the RSOS model near the (001) surface is BKT-rough.

### Surface velocity and “terrace slope”

Using the Monte Carlo method, the surface velocity is calculated by $V= [{\bar{h}}(t_0+\tau )-{\bar{h}}(t_0)]/\tilde{\tau }$, where ${\bar{h}}(t)$ is the mean surface height averaged over the surface area at time *t*, and $t_0$ and $\tilde{\tau }$ are $2\times 10^8$ MCS/site^62–64^.

At equilibrium, the terrace surface is exactly the (001) surface. However, at non-equilibrium, due to step-detachments the “terrace” surface is slightly tilted. The “terrace slope” is obtained from the mean-macrostep-height $\langle n \rangle$, which is calculated by the Monte Carlo method, as follows^62–64^:

16 $$\begin{aligned} p_1 = \sqrt{2} /\left( \frac{\sqrt{2}-{\bar{p}}}{{\bar{p}}z} +1\right) , \quad z = \frac{1}{\langle n \rangle }-\frac{N_m}{N_\text{step}}, \end{aligned}$$

where ${\bar{p}}$ is $N_\text{step}a/L$, $N_\text{step}$ is the number of elementary steps, and $N_m$ is the number of macrosteps in the simulated system.

## Supplementary Information

Supplementary Information.
