# Supplementary material for: Faceted-rough surface with disassembling of macrosteps in nucleation-limited crystal growth
Source: Sci Rep. 2021 Feb 12;11:3711. doi: 10.1038/s41598-021-83227-8 (PMC7881209; doi:10.1038/s41598-021-83227-8)
Supplement: Supplementary file 1 — Supplementary Information. [file 41598_2021_83227_MOESM1_ESM.pdf]

Faceted-rough surface with disassembling of macrosteps in nucleation-limited crystal growth

[1*]Noriko Akutsu
[1]*Faculty of Engineering, Osaka Electro-Communication University, Hatsu-cho, Neyagawa, Osaka 572-8530, Japan*

[1*] nori3@phys.osakac.ac.jp

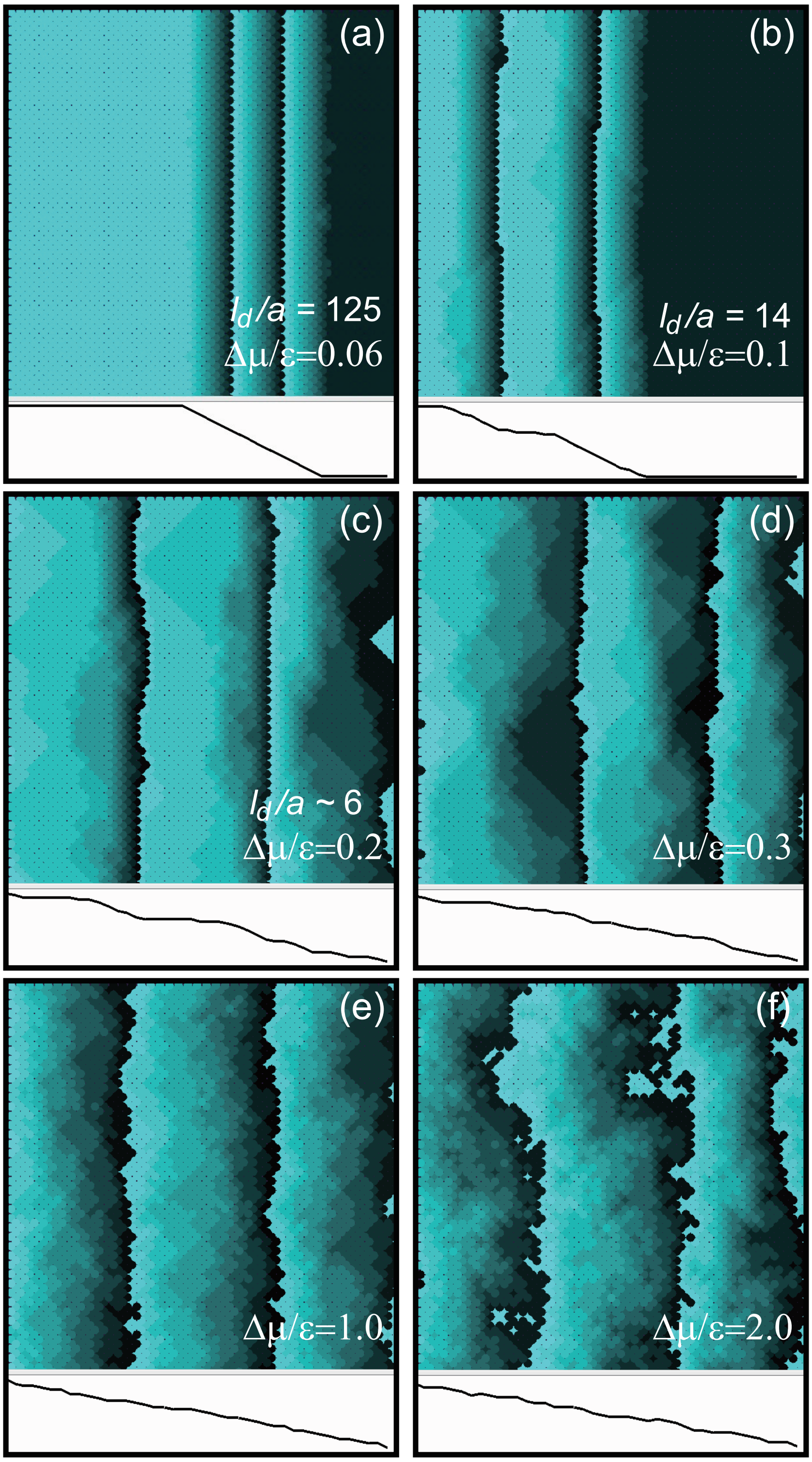

Figure S1. Snapshot of simulated surfaces at 4×10^8 MCS/site. (a) is smooth because $L < l_d/2$. (b) is faceted-rough. (c) and (d) are rough with locally faceted macrosteps. (e) is rough with $\alpha = 0.25$. (f) is KPZ-rough ($\alpha = 0.385$). Size: $40\sqrt{2} \times 40\sqrt{2}$. $N_{\rm step}$=30. $k_{\rm B}T/\epsilon = 0.4$. $\epsilon_{\rm int}/\epsilon = -0.9$. $p = N_{\rm step}a/L = 3\sqrt{2}/8 \approx 0.530$. $\theta = 27.9$ degrees. The surface height is represented by brightness with 10 gradations, where brighter regions are higher. Due to the finite gradation, where the darkest areas sit next to the brightest areas, the darker area is higher by one gradation unit. The lines of the side view are drawn with respect to the height along the bottom edge of the top-down view.

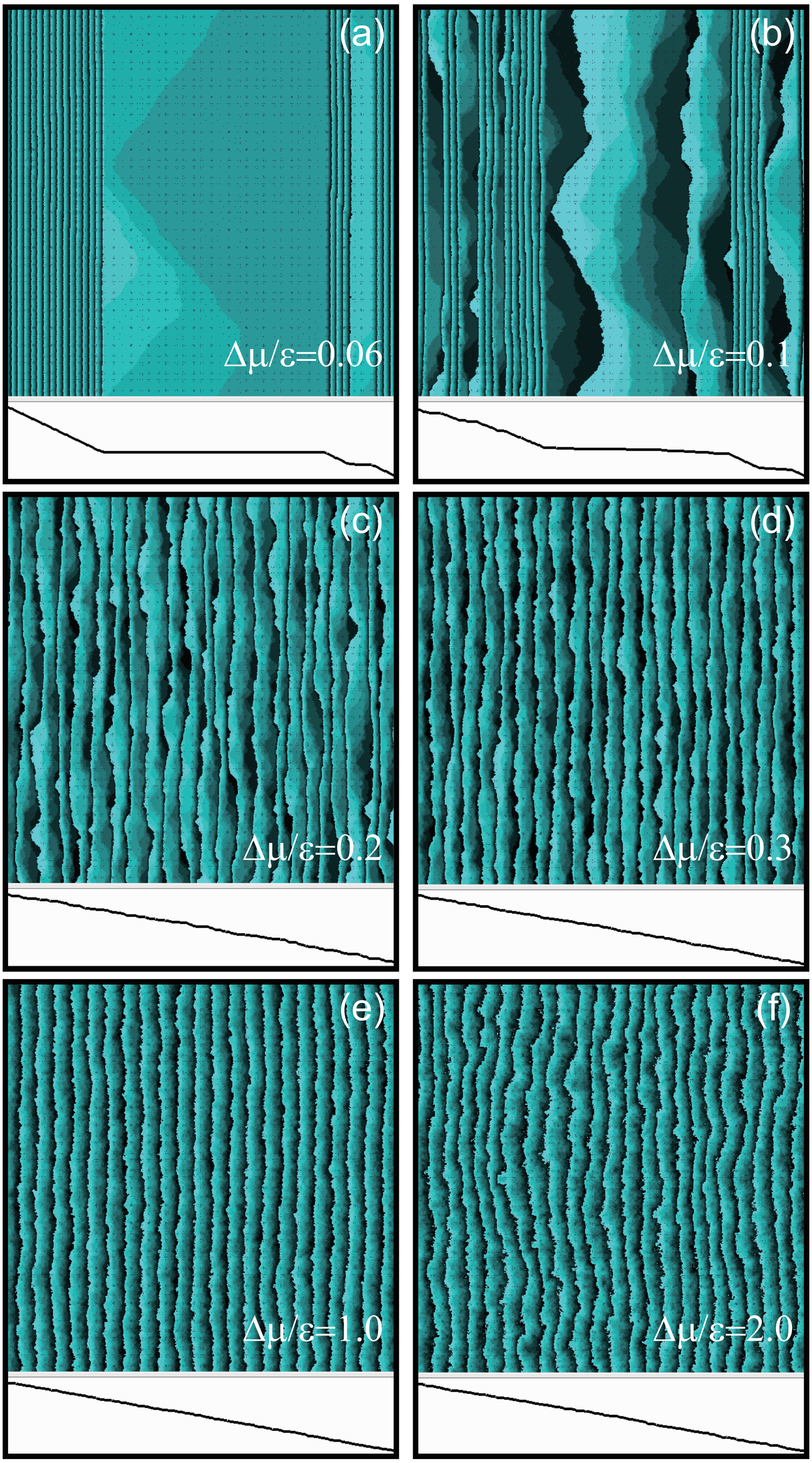

Figure S2. Snapshot of simulated surfaces at 4×10^8 MCS/site. (a), (b) are faceted-rough ($\alpha_p = 0.58 \sim 0.78$). (c), (d) are rough with locally faceted macrosteps. (e) is rough with $\alpha = 0.25$. (f) is KPZ-rough ($\alpha = 0.385$). Size: $320\sqrt{2} \times 320\sqrt{2}$. $N_{\rm step}$=240. $k_{\rm B}T/\epsilon = 0.4$. $\epsilon_{\rm int}/\epsilon = -0.9$. $p = N_{\rm step}a/L = 3\sqrt{2}/8 \approx 0.530$. $\theta = 27.9$ degrees.
